# Long Non-Coding RNA PCAT6 Induces M2 Polarization of Macrophages in Cholangiocarcinoma *via* Modulating miR-326 and RhoA-ROCK Signaling Pathway

**DOI:** 10.3389/fonc.2020.605877

**Published:** 2021-01-21

**Authors:** Jianfei Tu, Fazong Wu, Li Chen, Liyun Zheng, Yang Yang, Xihui Ying, Jingjing Song, Chunmiao Chen, Xianghua Hu, Zhongwei Zhao, Jiansong Ji

**Affiliations:** ^1^ Key Laboratory of Imaging Diagnosis and Minimally Invasive Intervention Research, the Fifth Affiliated Hospital of Wenzhou Medical University/Affiliated Lishui Hospital of Zhejiang University/Clinical College of The Affiliated Central Hospital of Lishui University, Lishui, China; ^2^ Department of Interventional Diagnosis and Treatment, The Central Hospital of Zhejiang Lishui, Lishui, China

**Keywords:** cholangiocarcinoma, PCAT6, miR-326, RhoA, macrophages, immune response

## Abstract

LncRNAs can act crucial roles in multiple tumors including cholangiocarcinoma (CCA). M2 polarization of macrophages is crucial for their biological roles in immunologic tolerance, which is able to induce tumorigenesis. Given that increasing evidence have suggested that lncRNAs could participate in modulating immune cell differentiation and function. Our current study was aimed to identify the underlying mechanism of lncRNA prostate cancer-associated transcript 6 (PCAT6) in CCA progression *via* regulating M2 macrophage polarization. PCAT6 has been reported as an oncogene in many cancers. In our work, we observed increased expression of PCAT6 in CCA patients. PCAT6 expression in various types of immune cells derived from CCA patients was tested by quantitative real-time PCR (qRT-PCR). It was revealed that PCAT6 was highly expressed in macrophages, which indicated that PCAT6 might regulate the function of macrophages to promote CCA progression. Then, *via* establishing CCA xenograft mouse model, we found loss of PCAT6 obviously triggered the immune response and reduced the *in vivo* tumor growth. In addition, overexpression of PCAT6 led to the M2 polarization of THP-1-differentiated macrophages. Moreover, miR-326 was predicted and proved as a target for PCAT6. In addition, down-regulation of PCAT6 repressed M2 polarization of macrophages, which was reversed by miR-326 inhibitors. The increase of PCAT6 induced the accumulation of ROS, mitochondrial and metabolic dysfunction in macrophages and mimics of miR-326 exhibited an opposite process. RohA has been recognized as a significant regulator of immune cell function. In our current work, we observed that RohA function as a downstream target for miR-326. In conclusion, our study highlighted a significant role of PCAT6/miR-326/RohA in immune response of macrophages in CCA and indicated PCAT6 as a potential target of immunotherapy in CCA.

## Introduction

Cholangiocarcinoma (CCA) is a frequent tumor of extrahepatic bile duct, which can extend from hilar area to bile duct (1). The etiology of CCA is related with cholelithiasis, sclerosing cholangitis and some other diseases (2). Although surgery, radiotherapy and chemotherapy have been widely be employed, the prognosis of CCA still remains poor (3, 4). Therefore, it is significant to explore effective therapies for diagnosis and treatment of CCA.

Tumor-associated macrophages are important immune cells within tumor micro-environment. They are closely associated with tumor angiogenesis and contributes to the worse prognosis (5). Tumor-associated macrophages are polarized into two phenotypes including M1 and M2 (6, 7). M1 polarized macrophages can secrete pro-inflammatory cytokines to remove tumor cells, while M2 polarized macrophages secrete anti-inflammation cytokines (8). Moreover, tumor-associated macrophages are considered to be the polarized M2 phenotype to trigger tumor progression (9).

LncRNAs are transcripts with over 200 bps with a limited protein-coding capacity (10). Increasing studies report lncRNAs are involved in various biological processes (11, 12). LncRNAs have recently drawn increasing attention because they can function as a ceRNA to hinder miRNA functions in tumors, including CCA (13). Furthermore, dozens of lncRNAs have been reported to participate in regulating macrophage polarization. For instance, lncRNA RPPH1 can induce colorectal cancer progression by interacting with TUBB3 to induce macrophage M2 polarization (14). XIST contributes to M2 polarization of macrophages in lung cancer (15). LncRNA PCAT6 has been reported to enhance CCA development by modulating miR-330-5p (16). However, the effect of PCAT6 on macrophage M2 polarization in CCA progression remains poorly known.

In our current work, we found that PCAT6 was increased in CCA patients and in macrophages derived from CCA patients. *Via* using orthotopic CCA mouse model, it was shown loss of PCAT6 repressed the immune response. This indicated PCAT6 may be involved in the immune tolerance of CCA. Subsequently, these data motivated us to investigate the function of PCAT6 in CCA *via* modulating macrophages.

## Methods and Materials

### Clinical Samples

Fresh CCA specimens and para-tumor tissues were collected from patients undergoing surgery in Affiliated Lishui Hospital of Zhejiang University. The histopathologic diagnosis was carried out by the pathologists based on WHO criteria. Peripheral whole blood of CCA patients were maintained in EDTA tubes before surgery. Our study was approved by the Institutional Ethics Committee of Affiliated Lishui Hospital of Zhejiang University. All the participants signed the written informed consent.

### Cell Culture

HuCCT1 and THP-1 cells were purchased from the Institute of Biochemistry and Cell Biology, Chinese Academy of Sciences (Shanghai, China). DMEM medium (HyClone Laboratories, Logan, UT, USA) with 10% FBS, 100 U/ml penicillin, and 100 μg/ml streptomycin was used to culture the cells. Cells were maintained at 37°C in a humidified incubator with 5% CO_2_. THP-1 cells were seeded and exposed to 320 nM PMA for 48 h to obtain macrophage-like differentiated THP-1 cells. Cells were cultured with 100 ng/ml IFN-γ for 48 h to generate M1 macrophages, while 20 ng/ml IL-4 was used to induce M2 macrophages. Peripheral blood mononuclear cells (PBMCs) were isolated from fresh blood samples of CCA patients by centrifugation over a Ficoll-Triyosom layer (Lymphoprep, Nycomed Pharma, Oslo, Norway), washed twice with saline and resuspended using complete medium (RPMI 1640 supplemented with 10% FBS, 100 U/ml penicillin, and 100 µg/ml streptomycin.

### Plasmid Construction and Lentivirus Infection

To over-express PCAT6, the ORF sequence of PCAT6 was cloned into pTracer-CMV2 vector (Jingmai BioTech, Chengdu, China). Then, cells were transfected using 2 μg PCAT6-OE or empty control vector using Lipofectamine 3000. To knockdown PCAT6, small hairpin sequence was cloned into pLKO.1 plasmid. PSPAX2-PMD2G system was used to package the lentivirus.

### Western Blotting Analysis

Cells were lysed using the RIPA buffer and protein concentration was tested using BCA protein assay kit. The proteins were separated by 10% sodium dodecyl sulfate polyacrylamide gel electrophoresis (SDS-PAGE) gel electrophoresis and then transferred onto a nitrocellulose membrane (Milipore, Billerica, MA, USA). After blocked using BSA, the membranes were incubated with primary antibodies against RohA, ROCK1, ROCK2, and GAPDH (Cambridge, MA, USA). The bands were indicated with goat anti-rabbit IgG-HRP secondary antibody (1:2,000; Abcam Cambridge, MA, USA) and were exposed using chemiluminescence substance (Thermo Fisher Scientific, Waltham, MA, USA).

### ELISA Assay

ELISA kits for the detection of IL-10, IL-6, IL-1β, IL-12, CD163, and Arg-1 levels were obtained from R&D systems (Minneapolis, MN, USA). The assay was carried out with all samples, standards, and controls assayed in duplicate. Subsequently, the absorbance was recorded using BioTek ELx800 (Thermo Fisher Scientific, Waltham, MA, USA).

### Immunofluorescence

Briefly, cells were seeded on the slides and fixed in 4% paraformaldehyde. Cells were permeabilized with PBS containing 0.1% Triton X-100. To block the samples, PBS containing 3% BSA was applied for 1 h. Then, the slides were incubated with prehybridization buffer at 40°C for 4 h and hybridized with digoxin-labeled probe for a whole night. Afterward, the slides were indicated with biotin conjugated anti-digoxin antibody. The samples were photographed using Zeiss Axio Imager Z1 fluorescence microscope.

### Immunochemistry

Tissue samples from the mice were fixed in 4% paraformaldehyde for a whole night. After dehydration and embedding, the samples were sliced into 5–8 μm thickness. The slices were stained with Ki-67 antibody overnight and with biotinylated secondary antibody. The sections were applied with DAB substrate and observed using microscopy (Zeiss, German).

### Flow Cytometry

To assess ROS levels in cells, 10μM DHE was added to the cells to carry out flow cytometry analysis. In order to test the uptake ability of glucose, 500μM 2-NBDG was added for 4 h. To evaluate the frequencies of IFN-γ+ in CD4+ or CD8+ T cells, the suspension tumor cells were stained using IFN-γ antibody (Abcam, followed by staining with fluorophore-conjugated secondary antibodies), and CD4 or CD8 antibody (eBioscience, San Diego, CA, USA). To assess the polarization of macrophages, 3×10^5^ cells were stained using CD11c, F4/80, CD11b, or CD206 antibodies (eBioscience, San Diego, CA, USA).

### Luciferase Reporter Assay

The sequence of WT of RohA and PCAT6 mRNA 3′-UTR and sequence of MUT after site-directed mutation of WT target site were synthesized. pGL3-RB-REPORT™ plasmid (RiboBio, Guangzhou, China) was digested by restriction endonuclease. Then the synthetic target gene fragments WT and MUT were inserted into pGL3-RB-REPORT™ vector. The vectors of MUT and WT were co-transferred to the cells with mimic-NC or miR-326 mimic. Luciferase Detection Kit (Beyotime, Shanghai, China) was used to determine the relative lights units.

### RNA Immunoprecipitation Assay

RIP was conducted using a Magna RIP RNA-Binding Protein Immunoprecipitation Kit (Millipore, Bedford, MA). Briefly, cell lysates were indicated with magnetic beads conjugated with negative control normal mouse IgG or human anti-Ago2 antibody (Millipore, Bedford, MA). Then, the immunoprecipitated RNAs were extracted and detected by qRT-PCR to confirm the enrichment of binding targets.

### Tumor Xenografts

Naïve CD8 + T cells from peripheral blood mononuclear cells (PBMCs) were isolated and then were purified. CCA-specific CD8 + T cells were stimulated using 1 mg/mL CD3 mAb, 5 mg/ml CD28 mAb, 20 ng/mL human rIL-2, 50 U/mL penicillin and 50 mg/Ml streptomycin. These naïve CD8 + T cells were then infected with shRNA of PCAT6 or LV-NC. Dendritic cells were differentiated from adherent monocytes in RPMI 1640 medium with IL-4 and GM-CSF. The obtained DCs were incubated using heat-shocked HuCCT1 cells to get antigen-loaded DCs (APCs). To obtain tumor antigen-specific CD8 + T cells, these treated naïve T cells were incubated with APCs for 3 days. 12 female BALB/c nude mice aged 4-6 weeks were obtained from the Animal research center of Chinese Academy of Sciences (Shanghai, China). Mice were then housed in specific pathogen-free units. 5×10^6^ HuCCT1 cells were mixed with Matrigel and subcutaneously injected in the right groin of the mice. To reconstitute human immune system, APC-stimulated naïve CD8 + T cells (pretreated with LV-shPCAT6 or LV-NC) were injected into the caudalmvein. One week later, the tumor weight of tumor blocks was measured using a vernier caliper and measurement was conducted every 3 days. All mice were sacrificed 22d after the surgery by cervical dislocation and the transplantation tumor was collected. All animal experiments were based on the Guide for the Care and Use of Laboratory Animals of the National Institutes of Health.

### qRT-PCR

RNA was extracted from CCA cells and tumor samples by TRIzol reagent (Thermo Fisher Scientific, Waltham, MA, USA) and RNeasy Plus Micro Kit (QIAGEN, Germantown, MD, USA). Reverse transcription was carried out to synthesize the Bestar qPCR RT Kit (DBI Bioscience, Shanghai, China). Quantitative RT-PCR was carried out in Applied Biosystems 7900 Real Time PCR System (Applied Biosystems, Foster City, CA, USA). Twenty nanograms of template in 25-µl reaction volume with 2×Power SYBR^®^ Green PCR Master Mix (Thermo Fisher Scientific, Waltham, MA, USA). The gene expression level for miR-326 was normalized to U6 RNA and mRNA expression was normalized to GAPDH expression using the comparative Ct method. Related primer sequences were provided in **Table 1**.

**Table 1:** Primers for real-time PCR.

Genes	Forward (5’-3’)	Reverse (5’-3’)
GAPDH	AGAAGGCTGGGGCTCATTTG	AGGGGCCATCCACAGTCTTC
PCAT6	CCCCTCCTTACTCTTGGACAAC	GACCGAATGAGGATGGAGACAC
miR-326	CATCTGTCTGTTGGGCTGGA	AGGAAGGGCCCAGAGGCG
RohA	GAGCCGGTGAAACCTGAAGA	TTCCCACGTCTAGCTTGCAG
U6	CTCGCTTCGGCAGCACA	AACGCTTCACGAATTTGCGT
ROCK1	AAAAATGGACAACCTGCTGC	GGCAGGAAAATCCAAATCAT
ROCK2	CGCTGATCCGAGACCCT	TTGTTTTTCCTCAAAGCAGGA
GLUT1	CAATGCTGATGATGAACCTG	GGGATGAAGATGATGCTCA
GLUT3	ATGGGGACACAGAAGGTCACC	AGCCACCAGTGACAGCCAAC
IL-6	GACTGATGTTGTTGACAGCCACTGC	AGCCACTCCTTCTGTGACTCTAACT
IL-1β	TCATGGGATGATGATGATAACCTGCT	CCCATACTTTAGGAAGACAGGGATTT
IL-12	CCACTCACATCTGCTGCTCAACAAG	ACTTCTCATAGTCCCTTTGGTCCAG
CD163	AGCAGACTACTCCAACATCC	TGGCACAGTTGTCTCTATCC
IL-10	GCCACCCTGATGTCTCAGTT	GTGGAGCAGGTGAAGAATGC
Arg-1	TGGA CAGACTAGGAATTGGCA	CCAGTCCGTCAACATCAAAACT

### Assessment of ATP Concentration and Oxygen Uptake Rates

Cellular ATP concentration was tested using the ATP detection kit (Beyotime, Shanghai, China). To measure oxygen uptake rates, MitoXpress Intra Kit (Luxcel Biosciences) was carried out.

### Statistical Analysis

Data was analyzed with Prism 6.0. Experiments were carried out in triplicates and the data was expressed as the means ± SD. One-way analysis of variance with multiple comparisons using Dunnett’s test was carried out for multiple comparison. *p* less than 0.05 was considered to be statistically significant.

## Results

### Expression of PCAT6 Was Significantly Increased in Cholangiocarcinoma Patients

**Figure 1:**
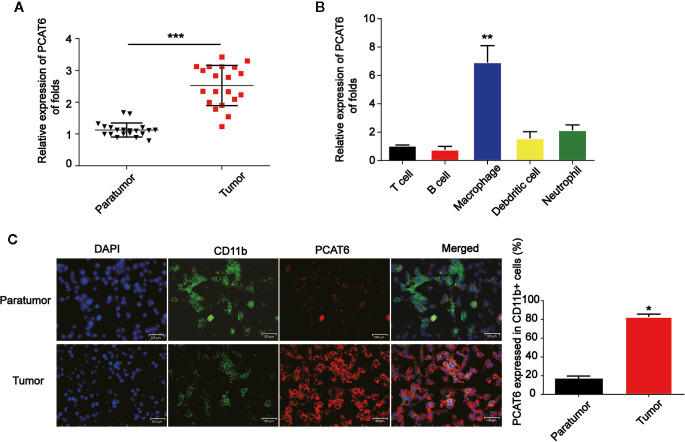
PCAT6 was elevated in cholangiocarcinoma (CCA) patients. **(A)** mRNA expression of PCAT6 in the tumor tissues of CCA patients (n=20) and the healthy tissues (n=20) were compared. **(B)** The expression of PCAT6 in patient-derived immune cells was assessed using qRT-PCR analysis. **(C)** The tissue sections of the paratumors and tumors from CCA patients were analyzed by IFSH. Scale bar, 100 μm. *P < 0.05; **P < 0.01; ***P < 0.001. Compared with the control group.

Firstly, to study the relationship between PCAT6 expression and the development of CCA, relative PCAT6 expression levels were obtained compared with non-tumor tissues (**Figure 1A**, n = 20 pairs). We observed that PCAT6 expression was highly increased in CCA tissues compared to the non-tumorous tissues. Further, we explored whether the high expression of PCAT6 participated in the immune response. It was shown that PCAT6 expression level was obviously expressed in the patient-derived in T cells, B cell, macrophages, dendritic cells, neutrophils (**Figure 1B**). The expression of PCAT6 was relatively higher in macrophages. Then, to confirm the expression of PCAT6 in tumor-associated immune cells, immunofluorescence analysis was carried out and we found that PCAT6 expression in CD11b+ cells within tumor tissues was greatly higher (**Figure 1C**). These findings suggested PCAT6 was relatively associated with tumor-associated macrophages in CCA.

### Loss of PCAT6 Inhibited the Progression of Cholangiocarcinoma via Activating T Cell Response *In Vivo*

**Figure 2:**
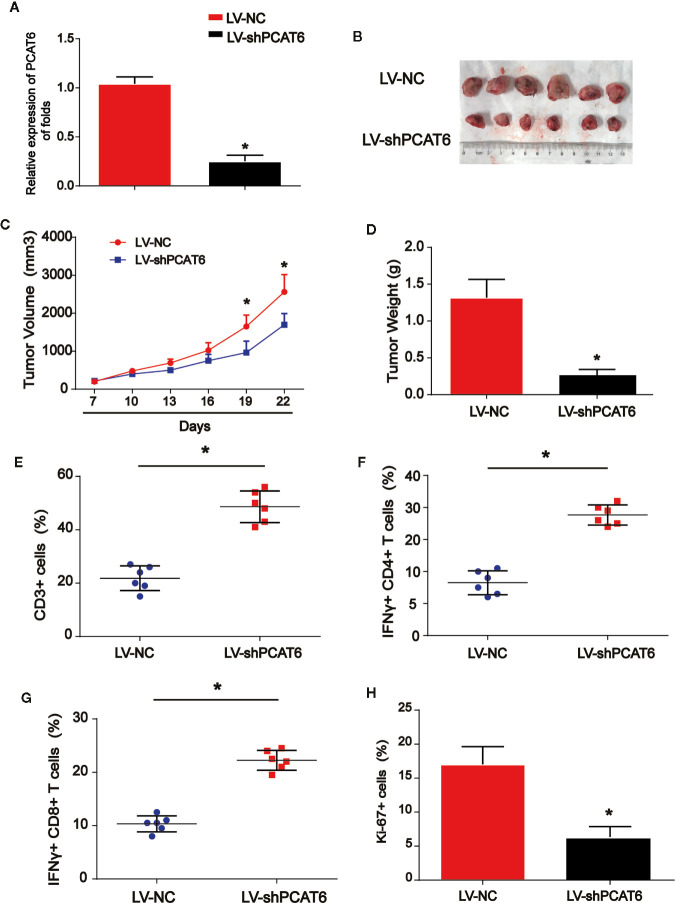
The down-regulation of PCAT6s activated the immune response *in vivo*. **(A)** The efficiency PCAT6 short hairpin RNA (shRNA) was confirmed using qRT-PCR analysis. **(B)** Nude mice with or without PCAT6 shRNA infected naïve CD8 + T cells incubated with CCA antigen-loaded DCs were injected with HuCCT1 cells. **(C)** Tumor growth curve. **(D)** Tumor weight. **(E)** Tumor sections were analyzed by IHC using CD3 antibody. **(F, G)** The frequencies of IFN-γ-producing CD4+ or CD8+ T cells in tumors of both groups were determined by flow cytometry analysis. **(H)** The tumor sections were analyzed by immunohistochemistry. *P < 0.05. Compared with the control group.

Next, to further explore the role of PCAT6 in immune cells in the tumorigenesis of CCA, HuCCT1 cells were subcutaneously injected in the right groin of the nude mice. LV-shPCAT6 or LV-NC infected naïve CD8 + T cells, which were incubated with CCA antigen-loaded DCs were transferred into these CCA tumor-bearing nude mice. The efficiency of PCAT6 shRNA was confirmed as displayed in **Figure 2A**. Then, the mice were injected by the cells subcutaneously. In **Figures 2B–D**, it was indicated that the growth of the tumors in PCAT6-shRNA group was inhibited than the control mice. Then, mice were sacrificed to study the phenotypes within tumor microenvironment. As shown in **Figure 2E**, the data displayed CD3+ cells in PCAT6-down-regulated mice tissues were significantly larger than the control mice. For another, it was implied that the percentage of IFN-γ-producing CD4+ and CD8+ cells in the tumors of PCAT6-down-regulated mice were significantly increased than the control mice (**Figures 2F, G**). In addition, immunochemistry analysis indicated that Ki-67+ cells in PCAT6 shRNA mice were significantly inhibited than the control mice (**Figure 2H**). These manifested the decrease of PCAT6 could enhance the T cell response *in vivo*.**Increase** in **PCAT6 Led to M2 Polarization of Macrophages**

**Figure 3:**
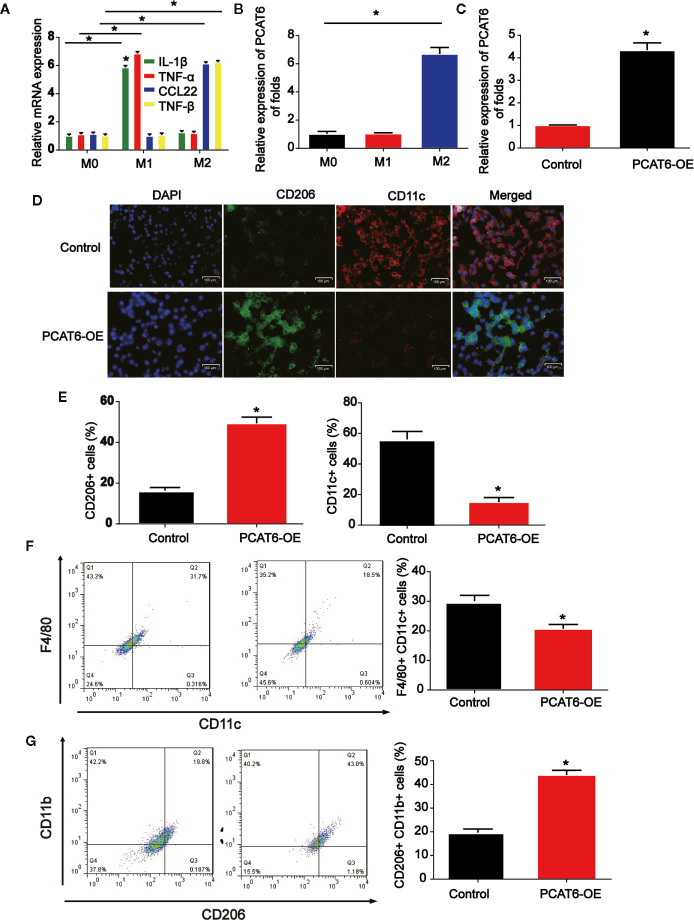
The effect of PCAT short hairpin RNA (shRNA) on M2-like polarization of macrophages. **(A)** Levels of M1 and M2-associated makers were determined in THP-1-diferentiated macrophages. **(B)** RNA was extracted from M1 and M2 and levels of lncRNA PCAT6 was determined using RT-qPCR in THP-1-diferentiated macrophages. **(C)** The efficiency PCAT6-OE was confirmed using qRT-PCR analysis. **(D, E)** Control and PCAT6-overexpressed macrophages were analyzed by immunofluorescence with CD206 and CD11c antibodies. Scale bar, 100 μm. **(F)** The control and PCAT6-overexpressed macrophages were analyzed by flow cytometry with M1 markers (CD11c and F4/80). **(G)** The control and PCAT6-overexpressed macrophages were analyzed with M2 markers (CD206 and CD11b). *P < 0.05. Compared with the control group.

To figure out the potential role of PCAT6, M1, and M2 macrophages were induced in THP-1-diferentiated macrophages. As indicated by the qRT-PCR data in **Figure 3A**, M1 and M2 polarized macrophages were triggered *in vitro* successfully. Next, we tested the mRNA expression level of PCAT6 in M1 and M2 cells. In **Figure 3B**, PCAT6 expression in M2 macrophages was obviously up-regulated than that of M1 macrophages. Then, THP-1-diferentiated macrophages were transfected with PCAT6-OE or the vector. In **Figure 3C**, PCAT6 was successfully induced by PCAT6-OE. Then, we observed that the expression of CD206 (M2 marker) in PCAT6-OE THP-1-differentiated macrophages. Cells were significantly up-regulated, whereas the expression of CD11c (M1 maker) was down-regulated (**Figures 3D, E**). It was presented that the ratio of F4/80+/CD11c cells were lower in the PCAT6-OE-transfected THP-1-differentiated macrophages in **Figure 3F**. The ratio of CD11b+/CD206+ cells in the PCAT6-OE-transfected THP-1-diferentiated macrophages was significantly induced as shown in **Figure 3G**.

### The Interaction Between PCAT6 and miR-326 in Macrophages

**Figure 4:**
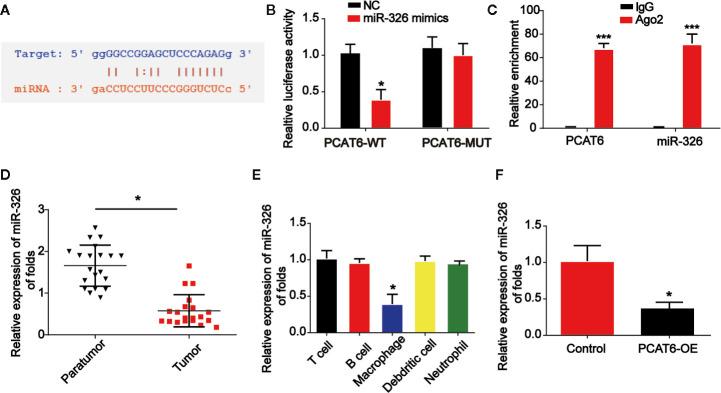
PCAT6 sponged miR-326. **(A)** The putative binding sites between PCAT6 and miR-326. **(B)** Luciferase activity was evaluated in macrophages co-transfected with PCAT6-WT or PCAT6-MUT reporter and miR-326 mimics in THP-1-differentiated macrophages. **(C)** RIP assays were performed using anti-Ago2 and IgG antibodies with extractions from THP-1-differentiated macrophages. **(D)** The expression of miR-326 in the tumor tissues of cholangiocarcinoma (CCA) patients. **(E)** The expression of miR-326 in different types of patient-derived immune cells was assessed using qRT-PCR analysis. **(F)** The expression of miR-326 in macrophages transfected with PCAT6 overexpression plasmid. *P < 0.05. Compared with the control group.

Online bioinformatics analysis STARBASE 2.0 (http://starbase.sysu.edu.cn/) was used and we predicted the miR-326 acted as a potential miRNA interacting with PCAT6 (**Figure 4A**). In addition, luciferase reporter vectors containing PCAT6-WT or PCAT6-MUT were constructed and co-transfected with miR-326 mimics or miR-NC into THP-1-differentiated macrophages. In **Figure 4B**, miR-326 repressed the luciferase activity of PCAT6-WT. In addition, RIP assays were applied in THP-1-differentiated macrophages. PCAT6 and miR-326 were obviously enriched by the anti-Ago2 antibody compared with the IgG antibody (**Figure 4C**). miR-326 expression level was reduced in CCA tissues (**Figure 4D**). Additionally, we found that PCAT6 expression level was obviously decreased in macrophages as indicated in **Figure 4E**. Overexpression of PCAT6 was able to reduce miR-326 expression *in vitro* significantly (**Figure 4F**).

### Loss of miR-326 Resulted in M2 Polarization of Macrophages

**Figure 5:**
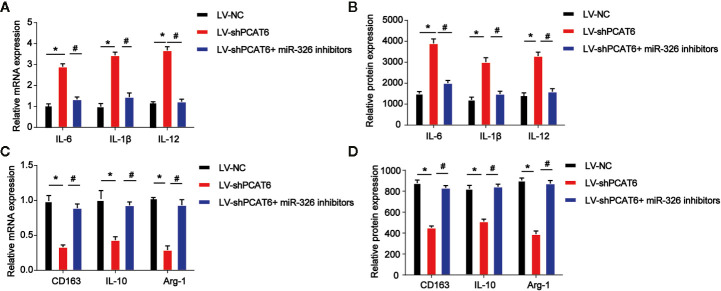
Effects of miR-326 on M2-like polarization of macrophages. THP-1-diferentiated macrophages were infected with PCAT6 short hairpin RNA (shRNA) and miR-326 inhibitors. **(A)** The messenger RNA (mRNA) expression levels of IL-6, IL-1β, and IL-12 were determined by qRT-PCR in THP-1-differentiated macrophages. **(B)** The protein expression levels of IL-6, IL-1β, and IL-12 were determined by ELISA assay in THP-1-differentiated macrophages. **(C)** The mRNA expression levels of IL-10, CD163 and Arg-1 were determined by qRT-PCR in macrophages. **(D)** The protein expression levels of IL-10, CD163, and Arg-1 in THP-1-diferentiated macrophages. *P < 0.05. Compared with the control group. ^#^P < 0.05. Compared with the LV-shPCAT6 group.

Then, THP-1-diferentiated macrophages were infected with PCAT6 shRNA and miR-326 inhibitors to assess the effect of miR-326 on M2 polarization of macrophages. In **Figures 5A, B**, the expression of three M1 macrophages specific marker genes, IL-6, IL-1β, and IL-12 were detected. mRNA and protein expression of IL-6, IL-1β, and IL-12 were increased by loss of PCAT6, which was reversed by the inhibitors of miR-326 (**Figures 5A, B**). Further, mRNA and protein expression levels of M2 marker, CD163, IL-10, and Arg-1 were found to be reduced by PCAT6 shRNA and miR-326 inhibitors enhanced their expression *in vitro* (**Figures 5C, D**).

### The Increase of PCAT6 Promoted Cellular Reactive Oxygen Species Production, Mitochondrial and Metabolic Dysfunction in Macrophages

**Figure 6:**
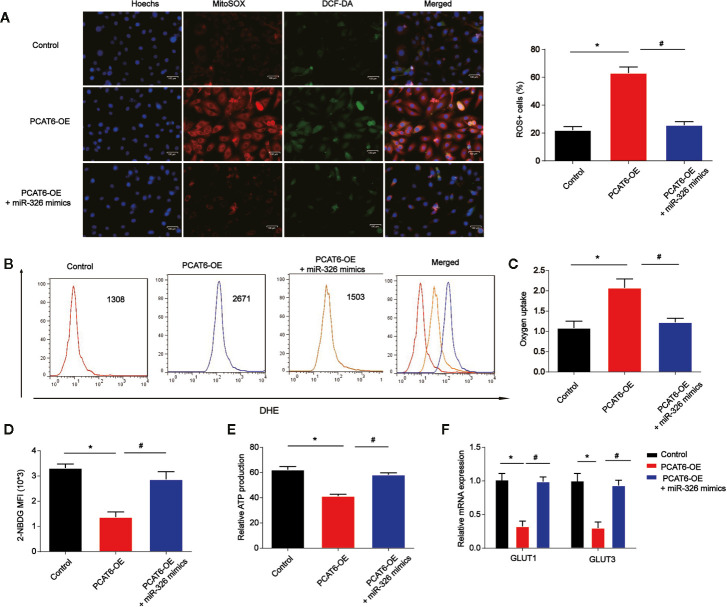
The overexpression of PCAT6 contributed to the accumulation of ROS, mitochondrial and metabolic dysfunction in macrophages. THP-1-diferentiated macrophages were infected with PCAT6 overexpression plasmid and miR-326 mimics. **(A)** Immunofluorescence with DCF-DA and MitoSOX staining were used to detect the levels of cytoplasmic and mitochondrial ROS. ROS+ cells were observed under microscopy. Scale bar, 100 μm. **(B)** The levels of DHE in macrophages were analyzed using flow cytometry. **(C)** Oxygen uptake rate in macrophages. **(D)** The levels of 2-NBDG were analyzed using flow cytometry in macrophages. **(E)** Relative ATP levels of the THP-1-diferentiated macrophages. **(F)** The messenger RNA (mRNA) levels of Glut1 and Glut3 were tested by qRT-PCR. *P < 0.05. Compared with the control group. ^#^P < 0.05. Compared with the PCAT6 OE group.

Moreover, to determine the role of PCAT6 and miR-326 in the ROS production in macrophages, the PCAT6-upregulated THP-1-diferentiated macrophages was constructed and we examined ROS levels with DCF-DA and mitoSOX probes. PCAT6-upregulated THP-1-diferentiated macrophages exhibited a higher ability of probe combination when compared with the control THP-1-diferentiated macrophages s, which was reversed by miR-326 mimics (**Figure 6A**). Furthermore, the DHE was used to detect the ROS levels *in vitro*. As shown, the DHE staining intensity of PCAT6-upregulated THP-1-diferentiated macrophages was significantly induced than the control THP-1-differentiated macrophages (**Figure 6B**). The oxygen uptake was increased by overexpression of PCAT6 while miR-326 mimics decreased that as exhibited in **Figure 6C**. We utilized 2-NBDG to assess the ability of cellular glucose uptake and proved that the incorporation of 2-NBDG in PCAT6-overexpressed THP-1-diferentiated macrophages was reduced and miR-326 overexpression increased 2-NBDG incorporation (**Figure 6D**). ATP production in PCAT6-overexpressed THP-1-diferentiated macrophages was lower (**Figure 6E**). The mRNA level of Glut1 and Glut3 was repressed by PCAT6 overexpression compared with the control cells as manifested in **Figure 6F**.

### RhoA Was a Direct Target of miR-326

**Figure 7:**
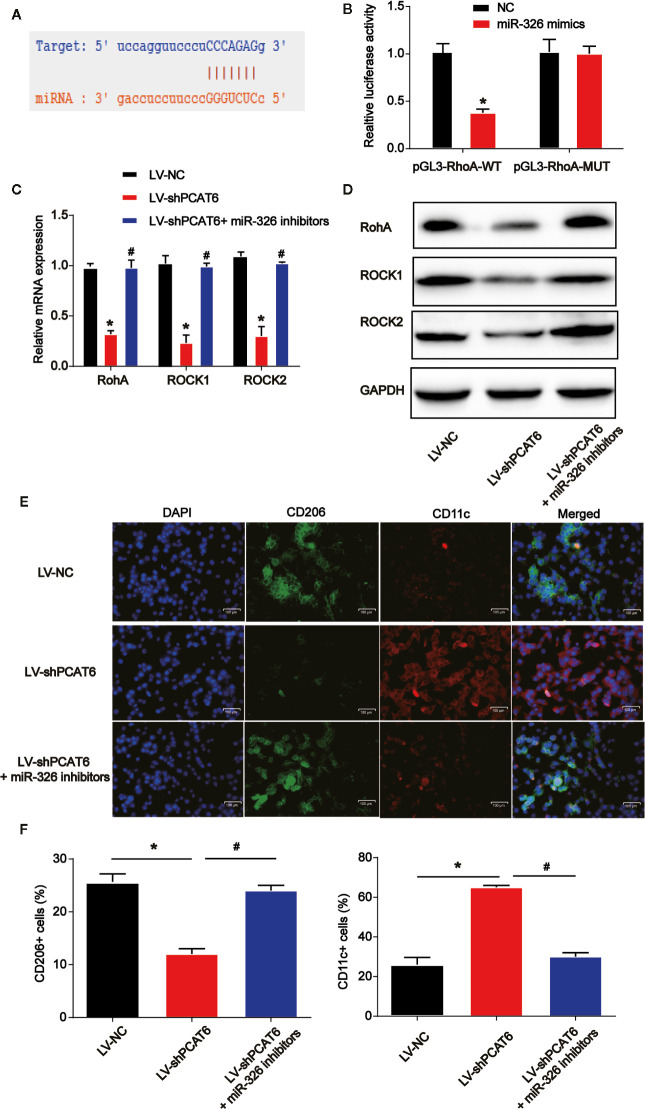
RhoA was a direct target of miR-326. **(A)** The putative binding sites between RhoA and miR-326. **(B)** Luciferase activity was evaluated in THP-1-diferentiated macrophages co-transfected with RhoA-WT or RhoA-MUT reporter and miR-326 mimics. **(C, D)** RhoA, ROCK1, and ROCK2 expression in THP-1-diferentiated macrophages transfected with PCAT6 short hairpin RNA (shRNA) and miR-326 inhibitors. **(E, F)** Immunofluorescence with M2 marker (CD206) and M1 marker (CD11b) was carried out. Scale bar, 100 μm. *P < 0.05. Compared with the control group. ^#^P < 0.05. Compared with LV-shPCAT6 group.

Next, we explored the downstream mechanism of miR-326 in modulating the immune responses of macrophages. RhoA was predicted as the target of miR-326 and the putative binding sites between then were indicated in **Figure 7A** *via* consulting STARBASE 2.0 (http://starbase.sysu.edu.cn/). Luciferase activity was evaluated in macrophages co-transfected with RhoA-WT or RhoA-MUT reporter and miR-326 mimics. In **Figure 7B**, overexpression of miR-326 obviously repressed the luciferase activity of RhoA-WT. Then, macrophages were infected with PCAT6 shRNA and miR-326 inhibitors. As exhibited in **Figures 7C, D**, loss of PCAT6 greatly reduced RhoA, ROCK1 and ROCK2 mRNA and protein expression in macrophages while miR-326 inhibitors reversed their expression levels. Then, in **Figures 7E, F**, immunofluorescence with M2 marker (CD206) and M1 marker (CD11b) was carried out. Down-regulation of PCAT6 reduced CD206+ cell ratio while induced CD11b+ cell ratio *via* modulating miR-326 in macrophages as shown in **Figures 7E, F**.

## Discussion

It has been well reported that lncRNA PCAT6 can act as an oncogene, which can drive the tumor progression in human cancers. For instance, PCAT6 can promote the development of gastric cancer *via* endogenously competing with miR-30 (17). PCAT6 can promote ovarian cancer occurrence and progression by inhibiting PTEN (18). In addition, PCAT6 can repress colon cancer cell apoptosis through regulating ARC expression *via* EZH2 (19). In this work, we observed PCAT6 was increased in CCA tissues and tumor-associated macrophages. Loss of PCAT6 reduced *in vivo* tumor growth *via* activating immune responses. In addition, overexpression of PCAT6 promoted M2 polarization of macrophages. Increase of PCAT6 promoted cellular ROS production, mitochondrial and metabolic dysfunction in macrophages *via* sponging miR-326 and regulating RohA/ROCK signaling.

Macrophages are highly plastic and they can exhibit different phenotypes such as proinflammatory M1 to anti-inflammatory M2 based on the environment (20). Tumor associated macrophages are present in high density in solid tumors sharing many characteristics with M2 macrophages. They have been identified to promote tumor development (21). Many lncRNAs are involved in M2 polarization of macrophages in cancers. For instance, loss of lncRNA SBF2-AS1in M2 macrophage-derived exosomes can elevate miR-122-5p to reduce XIAP and repress pancreatic cancer (22). GNAS-AS1 can induce ER+ breast cancer cell progression through inducing M2 macrophage polarization through modulating miR-433-3p and GATA3 (23). Here, we found that overexpression of PCAT6 induced M2 macrophage polarization in THP-1-differentiated macrophages. We proved that the ratio of M1 macrophages (F4/80+/CD11c cells) was significantly lower in the PCAT6-OE THP-1-differentiated macrophages while the ratio of M2 macrophages (CD11b+/CD206+ cells) in the PCAT6-OE THP-1-differentiated macrophages.

Then, we evaluated the potential mechanism of PCAT6 in regulating M2 macrophage polarization. miR-326 was predicted as a target for PCAT6. The previously identified miR-326 can participate in various cancers (24). For example, miR-326 can regulates endometrial cancer EMT and metastasis through targeting TWIST1 (25). miR-326 can act a tumor inhibitor in breast cancer through regulating ErbB/PI3K (26). The function of miR-326 in CCA progression remains poorly known. In our work, miR-326 was decreased in CCA tissues and overexpression of PCAT6 reduced miR-326 expression. In addition, miR-326 reversed the effect of PCAT6 on cellular ROS production, mitochondrial and metabolic dysfunction in THP-1-differentiated macrophages.

RhoA has been recognized as a member of small GTPase protein of Rho family (27). RhoA/ROCK is proved to be a crucial signaling pathway in tumor progression (28). RhoA can exhibit a crucial role in cancers and ROCK is an effector protein of RhoA. In addition, recent work has implicated aberrant RhoA/ROCK activation is involved in the pathogenesis of autoimmune disorders (29, 30). Targeting RhoA-ROCK pathway reverses T-cell dysfunction in SLE (31). SPON2 can enhance M1-like macrophage recruitment to repress hepatocellular carcinoma by regulating RhoA pathways (32). In addition, RhoA/ROCK has been linked with the progression of different cancers (33). RhoA was a downstream target of miR-326. Down-regulation of PCAT6 repressed RhoA/ROCK signaling *via* inducing miR-326 in macrophages. Many other mRNA targets of miR-326 were also predicted. In our future study, we would like to explore whether they are also involved in M2 polarization of macrophages in CCA.

In this study, PCAT6 was significantly increased in CCA patients and meanwhile, PCAT6 was highly expressed in macrophages. It was proved that PCAT6 participated in the immune response of CCA by modulating macrophages including ROS production, mitochondrial stress response, and M2 polarization through sponging miR-326 and activating RohA signaling. We highlighted PCAT6 might act as a potential target of immunotherapy for CCA treatment.

## Data Availability Statement

The raw data supporting the conclusions of this article will be made available by the authors, without undue reservation.

## Ethics Statement

The studies involving human participants were reviewed and approved by the Affiliated Lishui Hospital of Zhejiang University. The patients/participants provided their written informed consent to participate in this study. The animal study was reviewed and approved by the Affiliated Lishui Hospital of Zhejiang University.

## Author Contributions

JJ and ZZ contributed to the conceptualization and design of the study. JT and FW developed the methodology. LC, LZ, and YY contributed to the acquisition of data. XY and JS contributed to the analysis and interpretation of data. JT and FW contributed to the draft writing. JJ revised the manuscript. CC and XH provided administrative, technical, or material support. All authors contributed to the article and approved the submitted version.

## Funding

This study was supported by the Provincial and ministerial joint construction of key projects (grant No. WKJ-ZJ-1932), the Public welfare projects of Zhejiang Province (grant Nos. LGF19H180010, LGD19H160002, and LGF19H180009), and the Medical and Health Research Project of Zhejiang Province (grant Nos. 2018KY933).

## Conflict of Interest

The authors declare that the research was conducted in the absence of any commercial or financial relationships that could be construed as a potential conflict of interest.
