# Corrigendum: LncRNA PCAT6 Induces M2 Polarization of Macrophages in Cholangiocarcinoma via Modulating miR-326 and RhoA-ROCK Signaling Pathway

**DOI:** 10.3389/fonc.2021.680500

**Published:** 2021-04-22

**Authors:** Jianfei Tu, Fazong Wu, Li Chen, Liyun Zheng, Yang Yang, Xihui Ying, Jingjing Song, Chunmiao Chen, Xianghua Hu, Zhongwei Zhao, Jiansong Ji

**Affiliations:** ^1^ Key Laboratory of Imaging Diagnosis and Minimally Invasive Intervention Research, the Fifth Affiliated Hospital of Wenzhou Medical University/Affiliated Lishui Hospital of Zhejiang University/Clinical College of The Affiliated Central Hospital of Lishui University, Lishui, China; ^2^ Department of Interventional Diagnosis and Treatment, The Central Hospital of Zhejiang Lishui, Lishui, China

**Keywords:** cholangiocarcinoma, PCAT6, miR-326, RhoA, macrophages, immune response

In the original article, there was a mistake in the legend for ***Figure 2B*** as published. **Nude mice were injected with HuCCT1 cells with or without PCAT6 shRNA was described improperly**. The correct legend appears below.

**Nude mice with or without PCAT6 shRNA infected naïve CD8 + T cells incubated with CCA antigen-loaded DCs were injected with HuCCT1 cells.**

In the original article, there was an error. **The sentence “HuCCT1 cells were infected with shRNA of PCAT6 or the corresponding control” was described improperly**.

A correction has been made to **Results**, ***Loss of PCAT6 Inhibited the Progression of Cholangiocarcioma via Activating T Cell Response In Vivo***:

“HuCCT1 cells were subcutaneously injected in the right groin of the nude mice. LV-shPCAT6 or LV-NC infected naïve CD8 + T cells, which were incubated with CCA antigen-loaded DCs were transferred into these CCA tumor-bearing nude mice.”

In the original article, there was an error. **Some details were missing**.

A correction has been made to **Methods and Materials**, ***Tumor Xenografts***:

Naïve CD8 + T cells from peripheral blood mononuclear cells (PBMCs) were isolated and then were purified. CCA-specific CD8 + T cells were stimulated using 1 μg/mL CD3 mAb, 5 μg/ml CD28 mAb, 20 ng/mL human rIL-2, 50 U/mL penicillin and 50 mg/Ml streptomycin. These naïve CD8 + T cells were then infected with shRNA of PCAT6 or LV-NC. Dendritic cells were differentiated from adherent monocytes in RPMI 1640 medium with IL-4 and GM-CSF. The obtained DCs were incubated using heat-shocked HuCCT1 cells to get antigen-loaded DCs (APCs). To obtain tumor antigen-specific CD8 + T cells, these treated naïve T cells were incubated with APCs for 3 days. 12 female BALB/c nude mice aged 4-6 weeks were obtained from the Animal research center of Chinese Academy of Sciences (Shanghai, China). Mice were then housed in specific pathogen-free units. 5 × 106 HuCCT1 cells were mixed with Matrigel and subcutaneously injected in the right groin of the mice. To reconstitute human immune system, APC-stimulated naïve CD8 + T cells (pretreated with LV-shPCAT6 or LV-NC) were injected into the caudalmvein. One week later, the tumor weight of tumor blocks was measured using a vernier caliper and measurement was conducted every 3 days. All mice were sacrificed 22d after the surgery by cervical dislocation and the transplantation tumor was collected. All animal experiments were based on the Guide for the Care and Use of Laboratory Animals of the National Institutes of Health.”

The authors apologize for these errors and state that these do not change the scientific conclusions of the article in any way. The original article has been updated.

